# Genome sequence of the coffee root-knot nematode *Meloidogyne exigua*

**DOI:** 10.21307/jofnem-2021-065

**Published:** 2021-07-19

**Authors:** Ngan Thi Phan, Guillaume Besnard, Rania Ouazahrou, William Solano Sánchez, Lisa Gil, Sophie Manzi, Stéphane Bellafiore

**Affiliations:** 1 PHIM Plant Health Institute, University of Montpellier, IRD, CIRAD, INRAE, Institut Agro, Montpellier, France; 2 CNRS-UPS-IRD, UMR5174, EDB, 118 route de Narbonne, Université Paul Sabatier, 31062 Toulouse, France; 3 University of Rennes 1, UFR SVE, 35065, Rennes, France; 4 CATIE – Centro Agronómico Tropical de Investigación y Enseñanza, Turrialba, Costa Rica; 5 US 1426, GeT-PlaGe, Genotoul, INRAE, Castanet-Tolosan, France

**Keywords:** Genomics, Illumina, Mitogenome, Nanopore sequencing, Nuclear genome, Root-knot nematode

## Abstract

Root-knot nematodes (*Meloidogyne* spp.) cause serious damages on most crops. Here, we report a high-quality genome sequence of *Meloidogyne exigua* (population Mex1, Costa Rica), a major pathogen of coffee. Its mitogenome (20,974 bp) was first assembled and annotated. The nuclear genome was then constructed consisting of 206 contigs, with an N50 length of 1.89 Mb and a total assembly length of 42.1 Mb.

Root-knot nematodes (RKN) parasitize a wide range of host plants and have a global distribution. They are considered the most important group of plant-parasitic nematodes (Jones et al., 2013). Several *Meloidogyne* species can attack coffee plants, but only *Meloidogyne exigua* (Goeldi, 1892) has a significant impact on coffee production. This pathogen is the most widely distributed nematode in the coffee production areas in Central and South America (Campos and Villain, 2005), with estimated yield losses of up to 45% in the Rio de Janeiro State (Barbosa et al., 2004) and between 15 and 20% in Central America as a whole (Anzueto et al., 1995). Despite these serious impacts on coffee production, diversity and adaptation of *M. exigua* has been poorly documented, and so far, the only published study on the species was based on isozyme profiles and random amplified polymorphic DNA (RAPD) markers (Muniz et al., 2008). With the advent of high throughput sequencing methods, the analysis of its genome has become possible and may open new avenues for studying its evolutionary history.

Comparative genomics of RKN species has revealed a striking diversity in genome structure (e.g. chromosome counts, ploidy level, duplicated regions, heterozygosity) that might be linked to their different reproductive modes and species origin (Blanc-Mathieu et al., 2017; Castagnone-Sereno et al., 2013; Jaron et al., 2020; Triantaphyllou, 1985). Interestingly, despite prominent asexual reproduction in several RKN species, various mechanisms can generate genomic variability and may play a major role in their adaptability against different environments and hosts. These include, in particular, horizontal gene transfers (Danchin et al., 2016; Opperman et al., 2008; Phan et al., 2020), insertion of transposable elements (Kozlowski et al., 2020), and gene duplications/deletions (i.e. gene copy number variants; Castagnone-Sereno et al., 2019). *M. exigua* is a successful pathogen on coffee with a parthenogenetic reproduction mode (Triantaphyllou, 1985), and as demonstrated in other RKNs, its adaptation to various conditions may be also favored by above mentioned mechanisms. Here, we report a high-quality genome assembly of the genome of *M. exigua* population ‘Mex1’. The assembly represents a valuable molecular resource for future studies of phylogenomics on *Meloidogyne* species. In particular, this will foster comparative genomics to investigate and understand the evolutionary history of this nematode, the results of which may help in the development of new strategies for its management.

We used long-read Oxford Nanopore Technology (ONT) and short-read Illumina HiSeq sequencing data to generate the genome assembly. The population ‘Mex1’ was isolated from coffee roots collected in Hacienda Aquiares located in Turrialba, Cartago, Costa Rica (9°56′18.09′′N, 83°43′43.86′′W). A single juvenile was inoculated and multiplied on tomato (*Solanum lycopersicum* var. Moneymaker). The procedures for sequencing of *M. exigua* genome including nematode extraction and purification, genomic DNA extraction and purification, library preparation, and sequencing processes for the ONT and Illumina platforms were as described by Phan et al. (2020). For ONT sequencing, six mi`crograms of purified DNA were used to produce 3,150,177 raw reads with a total length of 15.16 Gb (N50 length = 13.9 kb; ca. 150-fold genome coverage). The ONT reads were trimmed to remove adapters using Porechop v.0.2.3 (Wick, 2019). Then, sequence was filtered for quality (*Q*-score ≥ 9) and length (*L* ≥ 500 bp) using NanoFilt v.1.1.0 (De Coster et al., 2018). Finally, 13.75 Gb of trimmed long reads (coverage of 137×) were selected for further analysis. Reads from the Illumina Technology were obtained from 3 µg of gDNA using the HiSeq3000 platform as described by Phan et al. (2020). Paired-end reads of 150 bp were generated (mean insert size = 452 bp), yielding 43.08 million reads (64.6  Gb; ca. 153-fold genome coverage). The quality of Illumina raw reads were assessed using FastQC (Andrews, 2010). Spades v.3.14.1 (Bankevich et al., 2012) and Blobtools v.2.1 (Kumar et al., 2013) were used to identify possible contamination; however, no potential contamination was detected. The Skewerv.0.2.2 software (Jiang et al., 2014) was used to trim reads using a minimum quality score of 30 and a minimum read length of 51 bp. Finally, the reads were error-corrected using Musket v.1.1 (Liu et al., 2013). Finally, 43.01 million trimmed pair-end reads (64.4 Mb,coverage of 152×) were used for the genome assembly.

The mitochondrial genome (mitogenome) of *M. exigua* was de novo assembled using short reads following the experimental procedure described by Besnard et al. (2014). Long reads were used to resolve the repeated sequences. A mitogenome sequence of 20,974 bp was constructed with an average coverage of 18,698×. Protein-coding genes and transfer RNAs (tRNAs) were annotated using the prediction pipeline of Mitos (Bernt et al., 2013; Donath et al., 2019) with the invertebrate mitochondrial code. Blastn search against the mitogenomes of *M. graminicola* (NC_024275.1) and *M. chitwoodii* (KJ476150) was used to confirm the prediction and to manually check the position of start/stop codons. Fourteen protein coding genes (*atp6*, *nad5*, *cox1*, *nad1*, *nad2*, *cox3*, *nad6*, *nad4L*, *cox2*, *rrnL*, *nad3*, *cob*, and *nad4*), two ribosomal RNA (rRNA) genes (*rrnS* and *rrnL*), two repeated regions (102 R and 313 R), and 21 transfer ribonucleic acid (tRNA) genes (*trnM*, *trnW*, *trnQ*, *trnA*, *trnR*, *trnV*, *trnE*, *trnS*, *trnT*, *trnY*, *trnL*_*2*_, *trnI*, *trnN*, *trnF*, *trnG*, *trnK*, *trnC*, *trnH*, *trnL*_*1*_, *trnP*, and *trnD*) were finally annotated from the mitogenome sequence. The mitogenome structure was visualized using the CIRCOS software (http://circos.ca/) (Fig. 1).

**Figure 1:**
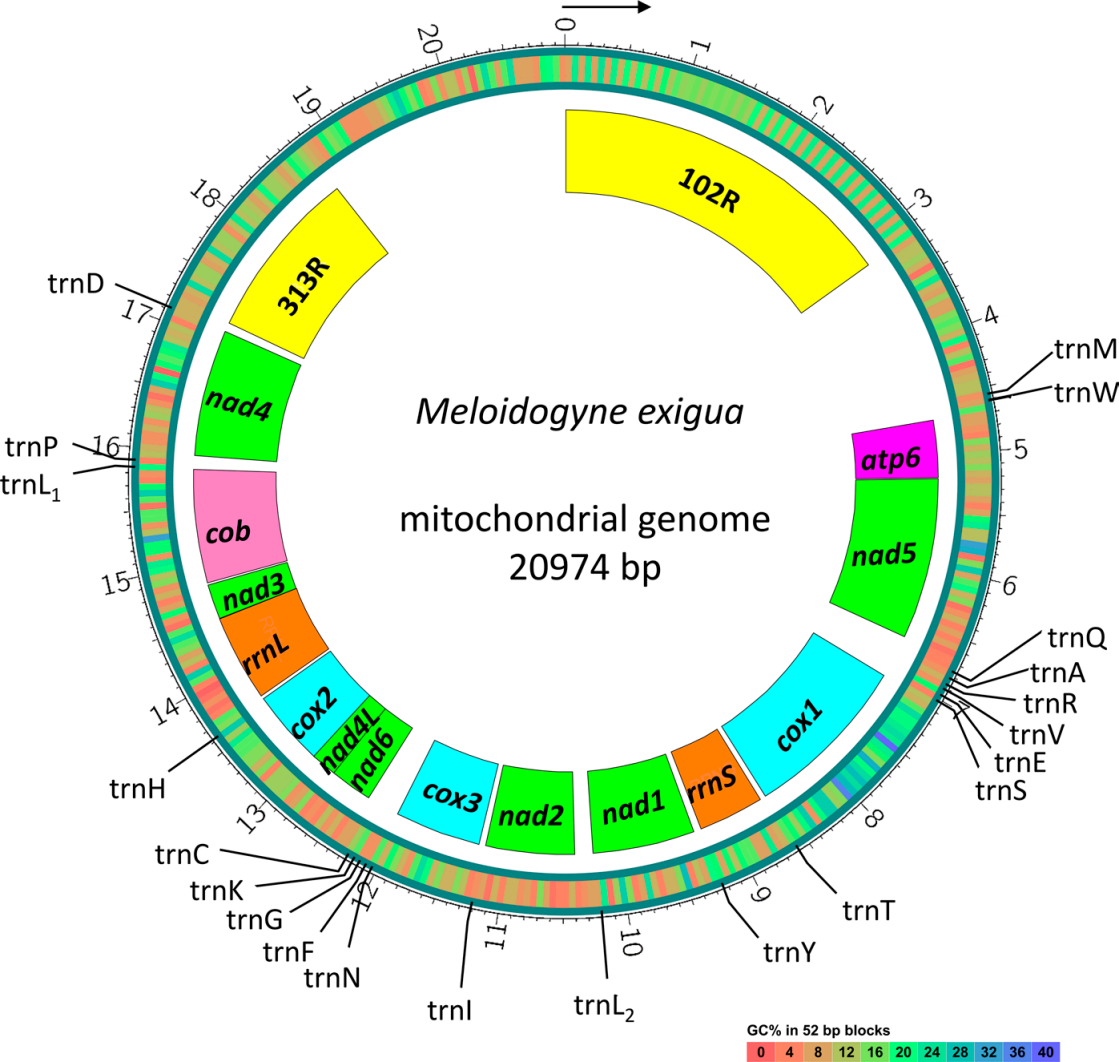
Circular gene map of the complete mitochondrial genome of *M. exigua*. Protein-coding genes, rRNA genes, and repeated regions are represented as boxes. Position of tRNAs are indicated by black lines. The direction of transcription of all genes is the same and indicated by the arrow. Abbreviations of protein coding and rRNA genes are: *nadi* = subunit i of NADH dehydrogenase; *coxi* = subunit i of cytochrome c oxydase; *cob* = cytochrome b; *atp6* = subunit 6 of ATP-synthase; *rrnS* = small subunit ribosomal RNA (12S); *rrnL* = large subunit ribosomal RNA (16S). tRNA genes (trnX) are named with a single-letter amino acid abbreviation (X) except for those coding for leucine, which are named as L_1_ (anticodon uag) and L_2_ (uaa). Two minisatellite regions, namely 102R and 313R, are composed of 102-bp and 313-bp repeats.

The reads that mapped to the mitogenome (with 100% identity; CIGAR = 100 M) were removed from the cleaned long and short reads datasets and the remaining sequences used for assembly of nuclear genome. The Canuv.1.8 software (Koren et al., 2017) was first used for the assembly. Subsequently, Racon v.1.4.3 (Vaser et al., 2017) and Pilon v.1.23 (Walker et al., 2014) were used to correct bases and homopolymer lengths. Contigs that had low read-coverage (<10×) were eliminated from the assembly to avoid artifacts and possible contamination. Finally, the assembled genome consisted of 206 contigs with a maximum contig length of 3,958 Kb and N50 of 1,882 Kb (Table 1). The total length of the assembly is 42.10 Mb, which matches the estimated haploid genome length of 43.2 Mb based on *k*-mer analyses (at *k* = 21) using Jellyfish v.1.0 (Marçais and Kingsford, 2011) and GenomeScope v.2.0 (Vurture et al., 2017) (Table 1 and Fig. 2A). Smudgeplot v.0.1.3 (Ranallo-Benavidez et al., 2020) and KMCv.3.0.0 softwares (Kokot et al., 2017) were used to estimate genome ploidy based on the *k*-mers counting (*k* = 21) of the short-read data. The genome is estimated to be diploid (AB) with heterozygosity of 0.03% (Fig. 2B). Blobtools (Laetsch and Blaxter, 2017) was used to assess contaminant DNA presence on the final genome assembly (Fig. 2C). Most of the genome assembly belong to Nematoda phylum (93.1%; Fig. 2C). One scaffold (1.18 Mb) was, however, assigned to the Arthopoda phylum (Fig. 2C). However, sequencing coverage and GC content of this scaffold were similar to other contigs of the genome assembly, and should thus be part of the nematode genome (Fig. 2C). The GC content of the assembled genome was 25.5% (Table 1).The Core Eukaryotic Genes Mapping Approach (CEGMA v.2.5) analysis (Parra et al., 2007) revealed that genome assembly contain 95.75% among 248 Eukaryotic Orthologs. The average number of orthologs per core gene at 1.09 indicated a haploid genome assembly. Besides, the genome assembly was 89.4% complete based on the eukaryote set (*n* = 303) of Benchmarking Universal Single-Copy Orthologs (BUSCO v.3.0.2) (Simão et al., 2015). Among available *Meloidogyne* genomes, this new assembly yields the second highest BUSCO completeness (after *Meloidogyne javanica*, summarized in Koutsovoulos et al., 2020) and the second largest N50 length (after *Meloidogyne chitwoodi*; Bali et al., 2021). This reference will assist a range of genetic, genomic, and phylogenetic studies to uncover diversity and evolution of *M. exigua* and other related RKNs.

**Table 1.** Statistics of the genome assemblyfor *M. exigua* obtained in our study (with Canu; Koren et al., 2017).

Assembly features	*M. exigua* genome
Total #scaffolds	260
Total length (bp)	42,101,073
Largest contig (bp)	3,958,915
N50 (bp)	1,882,513
N90 (bp	1,045,864
L50 (# scaffolds)	10
L90 (#scaffolds)	18
GC (%)	25.55
Mismatches	0
Gaps	0
CEGMA completeness^a^ (n:248)	C:95.97% (C+P: 97.18%)
BUSCO completeness^b^ (n:303)	C:89.4% [S: 89.1%, D: 0.3%]

**Notes:** ^a^C: Complete; C+P: Complete+Partial; ^b^C: Complete; S: Complete and single-copy; D: Complete and duplicated.

**Figure 2:**
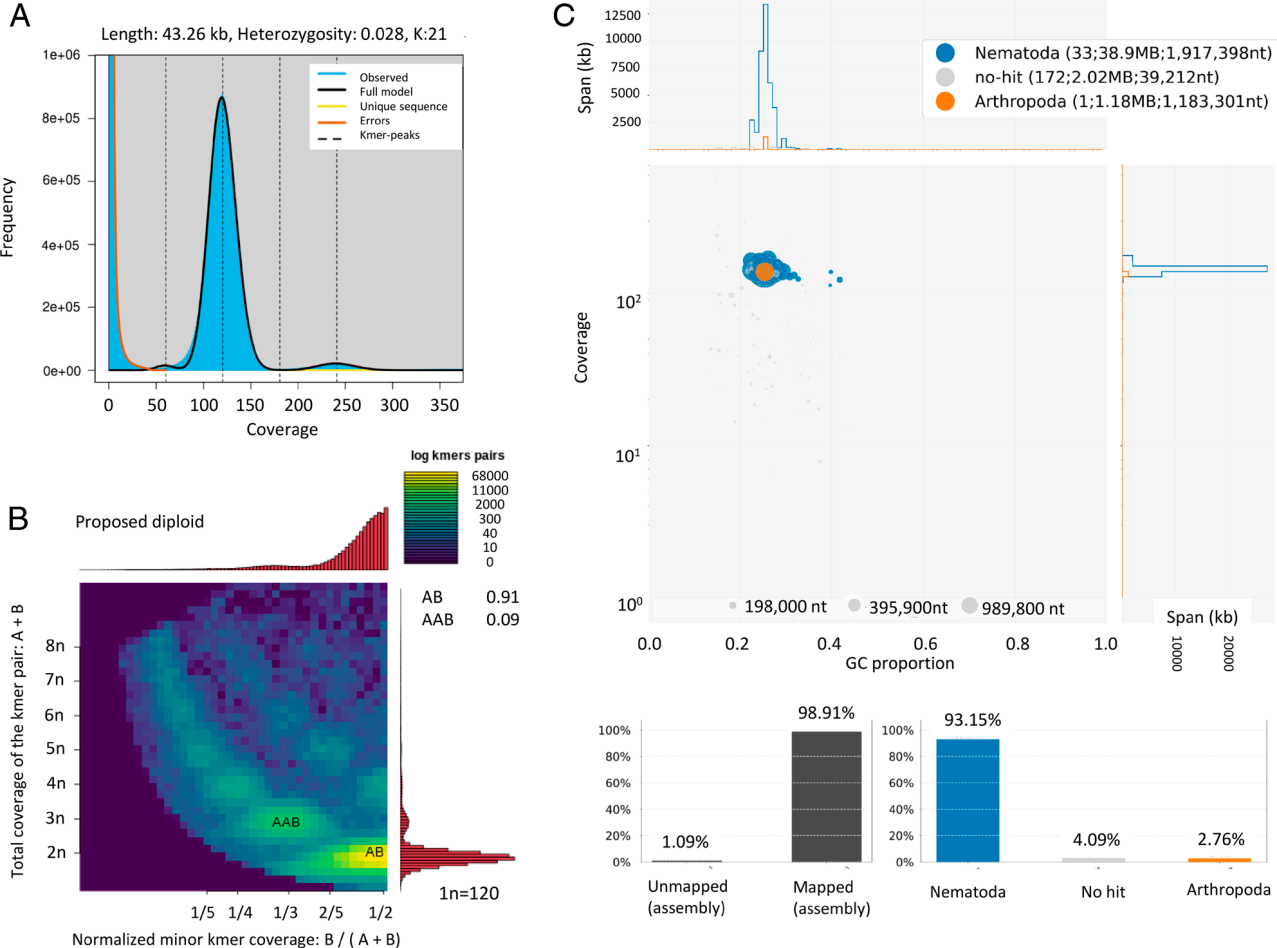
Haploid genome length, genome ploidy estimation and contaminant analysis of the *M. exigua* genome assembly. (A) GenomeScope profile showing estimated genome length of 43.26 Mb and heterozygosity of 0.028% at *k*-mer = 21. (B) Smudge plots showing the coverage and distribution of *k*-mer pairs that fit to diploid genome model. (C) Blobplot showing the lack of contamination in the final assembly by foreign (non-Nematoda) genetic material.

## Data availability and accession number(s)

Procedural information concerning the genome assembly and analysis presented in this paper can be found at the GitHub repository at https://github.com/PhanNgan/genome_assembly_mex. The mitogenome and nuclear genome sequences have been deposited in DDBJ/ENA/GenBankunder the accession numbers MZ359281 and JAGUQR000000000, respectively. The nuclear genome version described in this paper is version JAGUQR010000000.
